# Phylogenetic analysis suggests that sociality is associated with reduced effectiveness of selection

**DOI:** 10.1002/ece3.1886

**Published:** 2016-01-08

**Authors:** Virginia Settepani, Jesper Bechsgaard, Trine Bilde

**Affiliations:** ^1^ Department of BioscienceAarhus UniversityNy Munkegade 116, Building 15408000Aarhus CDenmark

**Keywords:** Effective population size, female biased sex‐ratio, inbreeding mating system, reproductive transition

## Abstract

The evolution of sociality in spiders is associated with female bias, reproductive skew and an inbreeding mating system, factors that cause a reduction in effective population size and increase effects of genetic drift. These factors act to decrease the effectiveness of selection, thereby increasing the fixation probability of deleterious mutations. Comparative studies of closely related species with contrasting social traits and mating systems provide the opportunity to test consequences of low effective population size on the effectiveness of selection empirically. We used phylogenetic analyses of three inbred social spider species and seven outcrossing subsocial species of the genus *Stegodyphus*, and compared dN/dS ratios and codon usage bias between social Inbreeding and subsocial outcrossing mating systems to assess the effectiveness of selection. The overall results do not differ significantly between the social inbreeding and outcrossing species, but suggest a tendency for lower codon usage bias and higher dN/dS ratios in the social inbreeding species compared with their outcrossing congeners. The differences in dN/dS ratio and codon usage bias between social and subsocial species are modest but consistent with theoretical expectations of reduced effectiveness of selection in species with relatively low effective population size. The modest differences are consistent with relatively recent evolution of social mating systems. Additionally, the short terminal branches and lack of speciation of the social lineages, together with low genetic diversity lend support for the transient state of permanent sociality in spiders.

## Introduction

The effective population size (Ne) affects the balance between effectiveness of selection and genetic drift, factors that are important in shaping the population dynamics of genes, and thereby adaptation. A reduction in effective population size is associated with an increase in the strength of genetic drift and thus lower effectiveness of selection (Wright 1931). Factors reducing effective population sizes include reproductive skew, biased sex ratio, and inbreeding (Frankham 1996; Leffler et al. 2012). Inbreeding populations may further experience demographic processes that affects effective population size such as recurrent bottlenecks (Schoen and Brown 1991), frequent cycles of extinction‐recolonization events (Charlesworth and Wright 2001), and low effective rate of recombination (Gordo and Charlesworth 2001). These processes may lead to a higher fixation probability of weakly deleterious mutations and loss of weakly advantageous mutations due to the stronger effect of drift (Charlesworth and Charlesworth 1987; Charlesworth 2003; Glémin and Galtier 2012). The genetic consequences of low effective population size may affect the evolutionary potential of species at different time scales. On a shorter timescale, strong drift acts to deplete genetic variation thereby reducing the adaptive potential based on standing genetic variation (Barrett and Schluter 2008). On a longer timescale, strong drift leads to a higher fixation probability of deleterious mutations. This results in accumulation of deleterious substitutions (“drift load”) over evolutionary time (Charlesworth and Wright 2001), which ultimately increases probability of extinction (Lynch et al. 1995).

Parameters frequently used to quantify the strength of drift and effectiveness selection are the ratio of non‐synonymous to synonymous substitutions (dN/dS ratio) and codon usage bias (CUB) (Cutter et al. 2008; Slotte et al. 2010; Qiu et al. 2011; Mattila et al. 2012). Reduced effectiveness of selection that result from low effective population size will cause a reduction in fixation probabilities of non‐synonymous adaptive mutations, and simultaneously in an increased probability of fixation of non‐synonymous deleterious mutations by drift. Because more deleterious than adaptive mutations are expected (Eyre‐Walker and Keightley 2007), the predicted net outcome is an increase in the rate of non‐synonymous substitutions leading to an increase in dN/dS ratio. Codon usage bias, i.e. the usage of synonymous codons that occur in different frequencies, is maintained by a balance between selection, mutation, and genetic drift. Selection acts on codon usage because certain codons are translated more accurately and/or efficiently than others (Hershberg and Petrov 2008). Codon preferences are determined by the mutational biases characteristic of each genome, however codon usage bias is expected to be stronger in highly expressed genes and in species characterized by a high effective population size. Reduced selection effectiveness is expected to result in the accumulation of less efficient codons and hence lower codon usage bias.

One approach to test predictions of Ne on the strength of drift and selection effectiveness is comparative studies of closely related species with contrasting mating systems that are expected to differ in effective population size (Cutter et al. 2008; Qiu et al. 2011). In plants, comparison of outcrossing and selfing sister species suggest that the effectiveness of selection is reduced in selfing species based on the findings of stronger codon usage bias in the outcrossing species (Qiu et al. 2011), and increased dN/dS ratios in selfing species (Glemin and Muyle 2014). In the nematode genera *Caenorhabditis* it has been shown that codon usage bias was equally strong in four outbreeding and two selfing species, which was interpreted as a recent evolutionary transition to selfing (Cutter et al. 2008). Additionally, the outcrossing *Caenorhabditis remanei* has been found to exhibit a ~20 fold higher genetic diversity than the self‐fertilizing congeners *C. elegans* and *C. briggsae* in orthologous loci (Graustein et al. 2002; Jovelin et al. 2003).

Here, we present a comparative study of the consequences of low Ne in a genus of sexually reproducing spiders that contains subsocial outcrossing and social inbreeding species. Evolution of sociality in spiders is associated with a number of characteristics, obligatory inbreeding through sib‐mating, highly female biased sex ratio (approximately 85% females) and reproductive skew (Lubin and Bilde 2007). These are all traits that act to reduce the effective population size, thereby increasing the effect of drift and decreasing the effectiveness of selection (Frankham 1996; Charlesworth and Wright 2001). Spider genera that contain multiple closely related species with contrasting life history and mating systems, outcrossing subsocial and permanently social species (Lubin and Bilde 2007), offer the opportunity to generate insights into the combined effects of reproductive skew, female bias and inbreeding mating systems that act to low effective populations size on the effectiveness of selection. The spider genus *Stegodyphus* consists of at least 20 species, and social inbreeding mating systems have originated three times independently (Kraus and Kraus 1988; Johannesen et al. 2007). A recent study suggests evidence for strong effects of drift on genetic diversity even at the species level in one of the social species *S. sarasinorum* (Settepani et al. 2014). Low genetic diversity and strong but shallow population differentiation in this social species indicates that high frequencies of population extinction and recolonization events result in homogenization of genetic diversity over the species' distribution range (Settepani et al. 2014), a dynamic process that decreases Ne. In contrast, subsocial congeners are characterized by equal sex ratio and premating dispersal which results in an outcrossing mating system (Bilde et al. 2005). Subsocial congeners are therefore expected to have a relatively higher effective population size which implies weaker effect of drift and stronger effectiveness of selection compared to their social sister species. Demographic modeling of RAD sequence data verifies these theoretical predictions, estimates of Ne demonstrates that three social *Stegodyphus* species have 10‐fold or lower effective population sizes compared to their subsocial sister‐species and 5–8 times lower estimates of genetic diversity. This is highly consistent over 3–5 populations per species, and therefore likely to result from their contrasting mating systems (Virginia Settepani et al. unpublished).

Here, we present a partial molecular phylogeny of the *Stegodyphus* genus including the three social species and their three subsocial sister species, and an additional four subsocial species. We examined predictions of the effect of low effective population size by contrasting dN/dS ratios and codon usage bias of species with inbreeding and outcrossing mating systems respectively. We asked the following question: do social inbreeding species experience reduced selection effectiveness compared with outcrossing subsocial congeners? If this is the case, we predict the social species to show increased dN/dS ratios and less optimized codon usage.

## Materials and Methods

### Species

We included 10 species from the genus *Stegodyphus* (Eresidae): three social species *S. sarasinorum*,* S. dumicola* and *S. mimosarum*, and seven subsocial species *S. lineatus*,* S. bicolor*,* S. dufuori*,* S. tibialis*,* S. mirandus*,* S. africanus* and *S. tentoriicola*. As outgroups we used *Eresus sandaliatus* (Stegodyphus' sister genus) and *Adonea fimbriata* (outgroup genus, also from Eresidae). We repeated our analyses independently with the inclusion of an undescribed *Stegodyphus* species recently discovered (for a total of 11 species from the genus *Stegodyphus*). Males and females of this potentially new species were found in Israel living in solitary nests in their adult stage, therefore we assume that this species is subsocial rather than social. Due to lack of knowledge about the biology and ecology of this species, we performed our analyses twice, both with and without this species included.

DNA was extracted from all samples using DNeasy Blood and Tissue Kit (Qiagen). In total, 13 randomly chosen nuclear loci were PCR amplified and sequenced from all samples by using primers custom designed from alignments of the social *S. mimosarum* and the subsocial *S. lineatus* and *S. tentoriicola* published in Mattila et al. (2012) (Table S1). The loci included 16 exons and 4 introns for a total of 5338 base pairs (of which 561 bp intronic).

### Data analyses

All chromatograms were manually inspected and wrongly called bases were corrected using Bioedit 7.0.4.1 (Hall 1999). Sequences were aligned using the clustalw algorithm implemented in Bioedit, followed by manual adjustment. Separate alignments were made for each locus. The loci were concatenated to estimate best species tree (Tonini et al. 2015) and the best substitution model for each exon and intron sequence was estimated with PartitionFinder (Lanfear et al. 2012) (Table S2).

The most likely tree topology was constructed using MrBayes 3.2 (Ronquist et al. 2012). MrBayes was run for 5 million generations, sampling frequency 500, burn‐in of 25% and two chains.

In order to confirm that the topology obtained was a true phylogenetic signal and not an artifact due to concatenation, we obtained an independent phylogeny for each partition. The most likely tree topology for each partition was constructed using MrBayes 3.2 (Ronquist et al. 2012) run for 5 million generations, sampling frequency 1000, burn‐in of 25% and two chains. The tree topologies constructed for each partition were consistent with the topology constructed for the concatenated sequences, supporting the robustness of the analysis.

We used DNAsp (Librado and Rozas 2009) to estimate the synonymous distance from present back to each node, and calculated the divergence time using the mutation rate of 8,4E‐08 estimated in Drosophila (Haag‐Liautard et al. 2007), under the assumption of a molecular clock. Synonymous distances to internal nodes were estimated by averaging over the estimates of all possible species pairs sharing the given nodes.

Codon usage biases (CUB) were estimated as Effective Number of Codons (ENC) for each species separately using INCA 1.2 (Supek and Vlahovicek 2004) using the concatenated alignment of all coding positions, and averages were calculated for social and subsocial species separately. 1000 new alignments were generated by bootstrapping over columns of codons. For each of those alignments, average ENC was estimated for social and subsocial species separately, and these estimates were used to obtain 95% confidence intervals. To test if CUB differed among subsocial and social species, the ENC estimates of the ten species were randomly divided into two groups of three and seven (corresponding to the number of social and subsocial species) by permutation, and the average from the group of seven was subtracted from the average of the group of three. This was done 1000 times to obtain a null distribution. To obtain a *P* value, the estimated difference between the average ENC of social and subsocial species was compared to this distribution. Base compositions were estimated for all three codon positions in all species, and were found to be highly similar (Fig. S1).

A maximum likelihood approach [PAML 4.6, (Yang 2007)] was used to estimate the rate of nonsynonymous (dN) to synonymous (dS) substitutions (dN/dS ratio). Before testing for the effect of mating system and life histories in social and subsocial species, we tested for the possible occurrence of sites under positive selection by using the “site models” implemented in PAML 4.6 (Yang 2007). We used a likelihood ratio test (LRT) with two degrees of freedom to compare model M7 (beta distribution of 0 ≤ x ≤ 1) and model M8 (beta distribution plus an extra class of sites with x > 1) (Glemin and Muyle 2014). These models were not significantly different from each other (*χ* ^2^ df = 2, *P* = 0.16) indicating the absence of sites under positive selection in the sequences, and therefore that the genes used mainly evolved under near‐neutrality. The same applies for the comparison of M7 and M8 for each partition separately.

We then estimated dN/dS ratios under four different models: (A) “*Single*”: dN/dS ratios were constrained to be identical for all branches in the tree; (B) “*Internal versus external*”: dN/dS ratios were constrained to be identical for internal branches and identical for external branches; (C) “*Internal versus external social versus external subsocial*”: dN/dS ratios were constrained to be identical for internal branches, for external branches leading to social species, and for external branches leading to subsocial species. This model assumes similar effective population sizes and therefore similar selection pressures for all subsocial species and for all social species respectively. Unpublished diversity estimates are consistent with such a scenario currently (Virginia Settepani et al., unpublished). Finally (D) “*Unconstrained*”: dN/dS ratios were estimated with no constrains, giving separate estimates for each branch in the tree. These models are nested, and likelihood ratio tests were used to evaluate which model fit the data best. The tree topology estimated by Mr. Bayes was used in the PAML analyses. All data analyses were repeated with the inclusion of an undescribed *Stegodyphus* sp. which was assumed to be subsocial, based on the finding of it living in solitary nests in the field similarly to other subsocial *Stegodyphus* (Y. Lubin, personal communication).

### Ethical note

The species used in this study are not subject to ethical laws in the country in which the sampling was performed.

## Results

**Figure 1:**
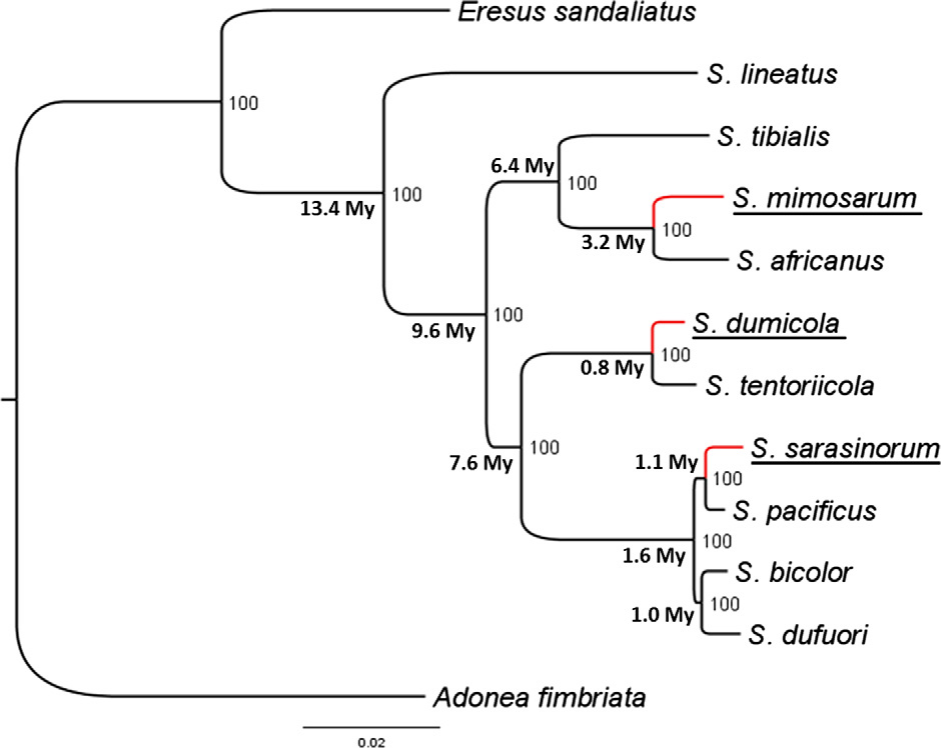
Bayesian phylogeny of the *Stegodyphus* genus. Posterior probabilities and divergence time estimates (in Million Years) are shown for each node. Red branched lead to social species which are underlined.

Sequences were obtained from all loci and all species except for one locus in each of the two outgroups (Table S1). We sequenced a total of 5338 bp of which 4749 bp coding and 589 bp non‐coding. All data were used to produce the phylogeny shown in Figure 1, but only coding data were used to estimate codon usage bias and dN/dS ratios. Posterior probabilities were 100 for all nodes providing the best possible support for the estimated topology (MrBayes convergence diagnostic: minimum ESS = 2901.8; mean ESS = 3028.2; average PSRF = 1).

Based on the mutation rate of 8,4E‐08 we estimated the *Stegodyphus* genus to be approximately 13 million years old. This is in accordance with the estimate in Mattila et al. (2012) assuming the same mutation rate. The estimated dates of all nine nodes can be seen in Figure 1. We note that the mutation rate used here may not reflect the actual mutation rates, and that a strict molecular clock model may be too simplistic, therefore, the time estimates should be taken with caution. We estimated all social lineages to be relatively young, 0.8, 1.1, and 3.2 million years old for *S. dumicola*,* S. sarasinorum,* and *S. mimosarum* respectively.

**Figure 2:**
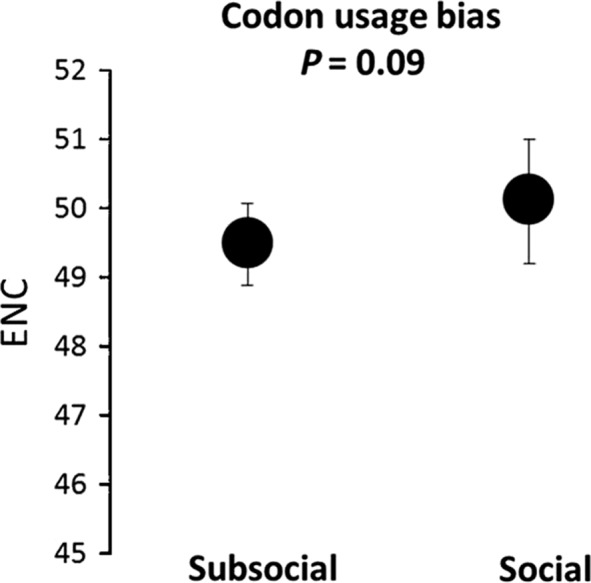
Codon usage biases estimates for subsocial and social species. ENC is the estimate of the effective number of codons.

Codon usage biases were estimated for each species as Effective Number of Codons (ENC). ENC values vary from 20 to 61: ENC = 20 represents extreme codon bias (only one codon is used for each amino acid) bias and ENC = 61 represents absence of codon usage bias (all codons are equally likely to code the amino acids). ENC values were slightly higher for the social (average ENC_soc_ = 50.13, lower and upper bound 49.20–50.99) compared to the subsocial species (average ENC_sub_ = 49.51, lower and upper bound 48.89–50.07), but this difference was not statistically significant (*P* = 0.09) (Fig. 2).

The results of the PAML analyses are summarized in Table 1. It is clear that adding free parameters (dN/dS) gives better fit to the data as the models “*Unconstrained”,* “*Internal versus external”* and “*Internal versus external social versus external subsocial*” all fit the data significantly better than the model “*Single”* (i.e. single dN/dS ratio for all species).

**Table 1:** Results of estimations rate of nonsynonymous (dN) to synonymous (dS) substitutions from four models: (A) *Single*: dN/dS ratios were constrained to be identical for all branches in the tree, (B) *Internal versus external branches*: dN/dS ratios were constrained to be identical for internal branches and identical for external branches, (C) *Internal versus external social versus external subsocial*: dN/dS ratios were constrained to be identical for internal branches, and separate estimates for external social branches and external subsocial branches, and (D) *Unconstrained external branches*: dN/dS ratios were estimated to be identical for internal branches and separate for all external branches

Model	−ln (likelihood)	dN/dS ratio	−2ΔlnL
A: Single	−9730.677616	0.1487	
B: Internal branches vs. external branches	−9728.514515	0.1675 (internal) 0.1214 (external)	A vs. B: 4.33 *P* < 0.05
C: Internal branches vs. external social branches vs. external subsocial branches	−9724.435196	0.1674 (internal) 0.2534 (external social) 0.1018 (external subsocial)	A vs. C: 12.48 *P* < 0.01 B vs. C: 8.16 *P* < 0.01
D: Unconstrained external branches	−9716.983258	0.0930 (*S. lineatus*) 0.0709 (*S. tibialis*) 0.1986 (*S. mimosarum* a) 0.1677 (*S. africanus*) 1.2489 (*S. dumicola* a) 0.2046 (*S. tentoriicola*) 0.0949 (*S. sarasinorum* a) 0.0958 (*S. pacificus*) 0.0359 (*S. bicolor*) 0.1445 (*S. dufuori*)	A vs. D: 27.39 *P* < 0.01 B vs. D: 23.06 *P* < 0.05 C vs. D: 14.90 NS

Results of Likelihood Ratio tests of nested models are shown in the last column.

a Social species.

Due to relative short external branches and the possibility of segregating polymorphisms inflating the dN/dS estimates (Paland and Lynch 2006; Mugal et al. 2014), we ran a model which separates estimates of dN/dS for internal and external branches. This model has a significantly better fit (*P* < 0.05) compared to the “*Single*” dN/dS model, with a lower external dN/dS ratio than internal dN/dS ratio (0.1214 vs. 0.1675). A third model with dN/dS ratio estimated separately for internal, external social, and external subsocial branches was used to test for the effect of social mating system. This model significantly improved the fit, and estimates a higher dN/dS ratio in the social branches compared to subsocial branches (0.2534 vs. 0.1018). The dN/dS estimate of the internal branches stayed unchanged.

Finally, the *“Unconstrained”* model, where dN/dS ratios were estimated with no constrains, revealed that dN/dS ratio estimates vary among social and subsocial species, therefore the assumption of similar dN/dS ratios for social and for subsocial species is not met. The social *S. dumicola* has a higher dN/dS ratio compared to other social species (1.2489 vs. 0. 0949 in *S. sarasinorum* and 0.1986 in *S. mimosarum*). To ensure that the results were not determined by the high dN/dS ratio of *S. dumicola*, we repeated the analysis grouping *S. dumicola* with all pairs of subsocial external branches separately instead of the three social species. This resulted in significantly elevated dN/dS ratio when *S. dumicola* was grouped with either *S. tentoriicola*,* S. africanus* or both. Finally, according to this last model, the subsocial *S. africanus* and *S. tentoriicola*, both sister species of a social species, have a higher dN/dS ratio (0.1677 and 0.2046, respectively) compared to other subsocial species in the genus (Table S3).

The inclusion of the undescribed *Stegodyphus* sp. did not change our results qualitatively for any parameter (Fig. S2).

## Discussion

Due to contrasting life history traits and demographic processes, we expect social species to have a lower effective population size compared to the effective population size of subsocial species, which should result in lower effectiveness of selection in social species. We used a phylogenetic approach to investigate whether dN/dS ratios and codon usage bias were different in social v*ersu*s subsocial species.

Our phylogenetic analysis of the *Stegodyphus* genus confirmed that the three social species evolved independently (Kraus and Kraus 1988; Johannesen et al. 2007). While previous studies were controversial regarding the monophyly or paraphyly of the *Stegodyphus* group (Johannesen et al. 2007; Miller et al. 2012), we show that *Stegodyphus* is a monophyletic group. Given the higher number of loci and the wider span of sequences applied here, we believe this is a reliable result.

In the inbred social species the dN/dS ratio was higher than the dN/dS ratio of outcrossed subsocial species. This finding corroborates our expectations of stronger drift and reduced effectiveness of selection on nonsynonymous mutations in the social species that are likely to experience relatively low Ne as a result of reproductive skew, strong female biased gender ratio, high nest‐ and population turnover dynamics and inbreeding mating system. It cannot be ruled out that evolution of sociality and regular inbreeding is associated with adaptive gene evolution, thereby increasing the dN/dS ratio in social species. However, adaptive mutations are extremely rare and an excess of deleterious mutations are expected (Eyre‐Walker and Keightley 2007), therefore this observed pattern is unlikely to be a result of positive selection. In addition, we find no sign of selection in the studied loci.

It is possible that segregating polymorphisms could inflate the dN/dS ratio in terminal branches (Paland and Lynch 2006; Glemin and Muyle 2014; Mugal et al. 2014). By running a model comparing the dN/dS ratio of internal and extrenal branches our results show that this was not the case in *Stegodyphus*, as the external dN/dS ratio were lower than internal branches. Very low levels of polymorphisms are segregating in the social species (Settepani et al. 2014). Estimates from RAD sequencing data suggest that social species are characterized by very low genetic diversity and 5–8 fold than that of subsocial species (Virginia Settepani et al., unpublished), suggesting that polymorphisms are not likely to strongly impact the dN/dS ratio estimates of the social species. Another potential reason for this result could be that *S. dumicola* alone drives this difference, since its estimated dN/dS ratio is very high. Grouping *S. dumicola* with all pairs of subsocial external branches separately instead of the three social species resulted in significantly elevated dN/dS ratio when *S. dumicola* was grouped with either *S. tentoriicola*,* S. africanus* or both. This suggests that the subsocial *S. tentoriicola* and *S. africanus*, also show sign of relaxed selection. Also, the different dN/dS ratios in social species suggest that the population dynamics characteristic in social species might have different intensities in the different species, alternatively that the times since the evolution of social mating systems differ. Overall, the elevated dN/dS ratio of social species provides indication of reduced effectiveness of selection in the genome of social spider species. However, according to the results of the “*Unconstrained”* model, it is evident that the assumption of similar dN/dS ratios for social and for subsocial species is not met. The dN/dS ratio estimates for the subsocial *S. tentoriicola* and *S. africanus* branches were relatively high compared to the other subsocial species in the genus (0.20 and 0.17 respectively). *Stegodyphus tentoriicola* and *S. africanus* are sister species of the social *S. dumicola* and *S. mimosarum*, leaving the possibility that an elevated dN/dS ratio is specific to certain clades rather than a result of a social life style. However, another interesting possibility is that the genus *Stegodyphus* contains species with different degree of social complexity: the sister species of social species might be characterized by life history traits and population dynamics which are closer to the ones experienced by social species rather than by the other subsocial species in the genus. An interesting study by Romiguier et al. (2014) demonstrated that dN/dS ratio in social insects (which also have a relatively low effective population sizes) is correlated with degree of social complexity: ant species with stronger worker‐queen dimorphism are characterized by higher dN/dS ratio. Our results show a comparable pattern: permanently social spider species appear to have the highest dN/dS ratio, followed by their subsocial sister species, followed by the other subsocial species in the genus. In agreement with this, the subsocial *S. tentoriicola* has occasionally been observed in the wild forming multifemale colonies (Y. Lubin and T. Bilde, personal communication). Additionally, extensive analyses of genomic diversity in social and subsocial species in this genus suggest the same pattern: social species have the lowest genetic diversity in the genus, followed by their sister species, followed by other subsocial species in the genus (Virginia Settepani et al., unpublished). Our results suggest that, similarly to what has been found in social insects (Romiguier et al. 2014), the degree of social complexity might be correlated with dN/dS ratio in genera containing social spider species. These results suggest that evolution of sociality that is associated with traits that reduce Ne is prone to suffer from relaxed effectiveness of selection and a build‐up of the genomic load.

Codon usage bias for each species, estimated as the effective number of codons (ENC), revealed higher average ENC, therefore lower codon usage bias, in the social inbred species compared with their subsocial outcrossed congeners, although the effect was not strong it indicates that selection on synonymous codons has been reduced in the social inbred species. Codon usage bias is a result of the combination of selection intensity and time, our result may therefore suggests that time since the transition to sociality was not sufficiently long for codon usage bias to build up.

Theory predicts that species with low effective population size may be subject to a build‐up of the genetic load and increased risk of extinction (Lynch et al. 1995), therefore selfing and inbreeding mating systems may be “evolutionary dead‐ends” (Stebbins 1957). Empirical tests of the genetic effects of low Ne comes mainly from comparative studies of selfing plants and outcrossing plants (Igic et al. 2008; Glemin and Muyle 2014) and show contrasting results. A study of 19 selfing and outcrossed species of grasses from the family Triticeae (Escobar et al. 2010), in *Arabidopsis* (Wright et al. 2002) and in six species of the nematode genus *Caenorhabditis* (Cutter et al. 2008) did not find evidence for reduced effectiveness of selection in codon usage bias and/or differences in rates of substitutions. On the other hand, a study by Slotte et al. (2010) showed differences in the effectiveness of selection between the outcrossing *Capsella grandiflora* and the selfing *Arabidopsis thaliana* that correlated well with their different life history traits and effective population sizes. Permanently social inbreeding spiders may similarly be evolutionary dead‐ends (Agnarsson et al. 2006; Johannesen et al. 2007). Our results suggest a buildup of genetic load in the social *Stegodyphus*, which may be linked with the level of social complexity of species, and which might result in lower adaptive potential, indicating that social spider species might represent an “evolutionary dead‐end” (Agnarsson et al. 2006). Phylogenetic analyses show that the social lineages of *Stegodyphus* and *Anelosimus* (a genus from the family *Theridiidae* that includes several independently evolved social species) form relatively short terminal and separate branches (Agnarsson et al. 2006; Johannesen et al. 2007). Phylogenetic analyses of plants show a pattern similar to that of social spiders, with selfing species mostly forming short terminal branches (Takebayashi and Morrell 2001). The combination of multiple independent origins of inbreeding sociality, low genetic diversity (Lubin and Bilde 2007; Settepani et al. 2014), short terminal branches, and no speciation of the social lineages lend support for the transient state of permanent sociality in spiders.

## Conclusions

We find that the genus *Stegodyphus* form a monophyletic group. Additionally, we detected a tendency toward difference in dN/dS ratio and codon usage bias between social and subsocial species which is modest but consistent with theoretical expectations of reduced effectiveness of selection in species with relatively low effective population size. Additionally the short terminal branches and lack of speciation of the social lineages, together with low genetic diversity lend support for the transient state of permanent sociality in spiders.

## Data Archiving

Sequence datasets supporting the results of this article will be available in the GenBank repository: accession numbers KU232626 ‐ KU232637, KU232638 ‐ KU232648, KU232649 ‐ KU232660, KU232661 ‐ KU232672, KU232673 ‐ KU232684, KU232685 ‐ KU232696, KU232697 ‐ KU232708, KU232709 ‐ KU232719, KU232720 ‐ KU232731, KU232732 ‐ KU232743, KU232744 ‐ KU232755, KU232756 ‐ KU232767, KU232768 ‐ KU232779.

## Conflict of Interest

The authors declare that they have no competing interests.

## Supporting information


**Table S1.** Primers designed for the phylogeny construction.
**Table S2.** Substitution models estimated in Partition Finder (Lanfear et al. 2012) for each locus.
**Table S3.** Inferred number of synonymous and non‐synonymous substitutions across each external branch.
**Figure S1.** Base composition estimates in social and subsocial species of the genus *Stegodyphus*.
**Figure S2.** Phylogeny including undescribed species *Stegodyphus* sp.

## References

[ece31886-bib-0001] Agnarsson, I. , L. Aviles , J. A. Coddington , and W. P. Maddison . 2006 Sociality in Theridiid spiders: repeated origins of an evolutionary dead end. Evolution 60:2342–2351. 17236425

[ece31886-bib-0002] Barrett, R. D. H. , and D. Schluter . 2008 Adaptation from standing genetic variation. Trends Ecol. Evol. 23:38–44. 18006185 10.1016/j.tree.2007.09.008

[ece31886-bib-0003] Bilde, T. , Y. Lubin , D. Smith , J. M. Schneider , and A. A. Maklakov . 2005 The transition to social inbred mating systems in spiders: role of inbreeding tolerance in a subsocial predecessor. Evolution 59:160–174. 15792236

[ece31886-bib-0004] Charlesworth, D. 2003 Effects of inbreeding on the genetic diversity of population. Philos. Trans. R. Soc. Lond. B Biol. Sci. 358:1051–1070. 12831472 10.1098/rstb.2003.1296 PMC1693193

[ece31886-bib-0005] Charlesworth, D. , and B. Charlesworth . 1987 Inbreeding depression and its evolutionary consequences. Annu. Rev. Ecol. Syst. 18:237–268.

[ece31886-bib-0006] Charlesworth, D. , and S. I. Wright . 2001 Breeding systems and genome evolution. Curr. Opin. Genet. Dev. 11:685–690. 11682314 10.1016/s0959-437x(00)00254-9

[ece31886-bib-0007] Cutter, A. D. , J. D. Wasmuth , and N. L. Washington . 2008 Patterns of molecular evolution in caenorhabditis preclude ancient origins of selfing. Genetics 178:2093–2104. 18430935 10.1534/genetics.107.085787 PMC2323799

[ece31886-bib-0008] Escobar, J. S. , A. Cenci , J. Bolognini , A. Haudry , S. Laurent , J. David , et al. 2010 An integrative test of the dead‐end hypothesis of selfing evolution in Triticeae (Poaceae). Evolution 64:2855–2872. 20500214 10.1111/j.1558-5646.2010.01045.x

[ece31886-bib-0009] Eyre‐Walker, A. , and P. D. Keightley . 2007 The distribution of fitness effects of new mutations. Nat. Rev. Genet. 8:610–618. 17637733 10.1038/nrg2146

[ece31886-bib-0010] Frankham, R. 1996 Relationship of genetic variation to population size in wildlife. Conserv. Biol. 10:1500–1508.

[ece31886-bib-0011] Glémin, S. , and N. Galtier . 2012 Genome evolution in outcrossing versus selfing versus asexual species. Methods Mol. Biol. 855:311–335. 22407714 10.1007/978-1-61779-582-4_11

[ece31886-bib-0012] Glemin, S. , and A. Muyle . 2014 Mating systems and selection efficacy: a test using chloroplastic sequence data in Angiosperms. J. Evol. Biol. 27:1386–1399. 24674012 10.1111/jeb.12356

[ece31886-bib-0013] Gordo, I. , and B. Charlesworth . 2001 Genetic linkage and molecular evolution. Curr. Biol. 11:R684–R686. 11553339 10.1016/s0960-9822(01)00408-0

[ece31886-bib-0014] Graustein, A. , J. M. Gaspar , J. R. Walters , and M. F. Palopoli . 2002 Levels of DNA polymorphism vary with mating system in the nematode genus Caenorhabditis. Genetics 161:99–107. 12019226 10.1093/genetics/161.1.99 PMC1462083

[ece31886-bib-0015] Haag‐Liautard, C. , M. Dorris , X. Maside , S. Macaskill , D. L. Halligan , B. Charlesworth , et al. 2007 Direct estimation of per nucleotide and genomic deleterious mutation rates in Drosophila. Nature 445:82–85. 17203060 10.1038/nature05388

[ece31886-bib-0016] Hall, T. A. 1999 BioEdit: a user‐friendly biological sequence alignment editor and analysis program for Windows 95/98/NT. Nucleic Acids Symp. Ser. 41:95–98.

[ece31886-bib-0017] Hershberg, R. , and D. A. Petrov . 2008 Selection on codon bias. Annu. Rev. Genet. 42:287–299. 18983258 10.1146/annurev.genet.42.110807.091442

[ece31886-bib-0018] Igic, B. , R. Lande , and J. R. Kohn . 2008 Loss of self‐incompatibility and its evolutionary consequences. Int. J. Plant Sci. 169:93–104.

[ece31886-bib-0019] Johannesen, J. , Y. Lubin , D. R. Smith , T. Bilde , and J. M. Schneider . 2007 The age and evolution of sociality in Stegodyphus spiders: a molecular phylogenetic perspective. Proc. R. Soc. Lond. B Biol. Sci. 274:231–237. 10.1098/rspb.2006.3699 PMC1685853 17148252

[ece31886-bib-0020] Jovelin, R. , B. C. Ajie , and P. C. Phillips . 2003 Molecular evolution and quantitative variation for chemosensory behaviour in the nematode genus Caenorhabditis. Mol. Ecol. 12:1325–1337. 12694294 10.1046/j.1365-294x.2003.01805.x

[ece31886-bib-0021] Kraus, O. , and M. Kraus . 1988 The genus *Stegodyphus* (Arachnida, Araneae). Sibling species, species groups, and parallel origin of social living Pp. 151–254 *in* Kraus O., ed. Verhandlungen des Naturwissenschaftlichen Vereins in Hamburg (NF) 30. Verlag Paul Parey, Hamburg and Berlin.

[ece31886-bib-0022] Lanfear, R. , B. Calcott , S. Y. W. Ho , and S. Guindon . 2012 PartitionFinder: combined selection of partitioning schemes and substitution models for phylogenetic analyses. Mol. Biol. Evol. 29:1695–1701. 22319168 10.1093/molbev/mss020

[ece31886-bib-0023] Leffler, E. M. , K. Bullaughey , D. R. Matute , W. K. Meyer , L. Ségurel , A. Venkat , et al. 2012 Revisiting an old riddle: what determines genetic diversity levels within species? PLoS Biol. 10:e1001388. 22984349 10.1371/journal.pbio.1001388 PMC3439417

[ece31886-bib-0024] Librado, P. , and J. Rozas . 2009 DnaSP v5: a software for comprehensive analysis of DNA polymorphism data. Bioinformatics 25:1451–1452. 19346325 10.1093/bioinformatics/btp187

[ece31886-bib-0025] Lubin, Y. , and T. Bilde . 2007 The evolution of sociality in spiders. Adv. Study Behav. 37:83–145.

[ece31886-bib-0026] Lynch, M. , J. Conery , and R. Burger . 1995 Mutation accumulation and the extinction of small populations. Am. Nat. 146:489–518.

[ece31886-bib-0027] Mattila, T. M. , J. S. Bechsgaard , T. T. Hansen , M. H. Schierup , and T. Bilde . 2012 Orthologous genes identified by transcriptome sequencing in the spider genus Stegodyphus. BMC Genom. 13:70. 10.1186/1471-2164-13-70 PMC3350440 22333217

[ece31886-bib-0028] Miller, J. A. , C. E. Griswold , N. Scharff , M. Rezac , T. Szuts , and M. Marhabaie . 2012 The velvet spiders: an atlas of the Eresidae (Arachnida, Araneae). ZooKeys 195:1–144. 22679386 10.3897/zookeys.195.2342 PMC3361087

[ece31886-bib-0029] Mugal, C. F. , J. B. W. Wolf , and I. Kaj . 2014 Why time matters: codon evolution and the temporal dynamics of dN/dS. Mol. Biol. Evol. 31:212–231. 24129904 10.1093/molbev/mst192 PMC3879453

[ece31886-bib-0030] Paland, S. , and M. Lynch . 2006 Transitions to asexuality result in excess amino acid substitutions. Science 311:990–992. 16484491 10.1126/science.1118152

[ece31886-bib-0031] Qiu, S. , K. Zeng , T. Slotte , S. Wright , and D. Charlesworth . 2011 Reduced efficacy of natural selection on codon usage bias in selfing Arabidopsis and Capsella species. Genome Biol. Evol. 3:868–880. 21856647 10.1093/gbe/evr085 PMC3296465

[ece31886-bib-0032] Romiguier, J. , J. Lourenco , P. Gayral , N. Faivre , L. A. Weinert , S. Ravel , et al. 2014 Population genomics of eusocial insects: the costs of a vertebrate‐like effective population size. J. Evol. Biol. 27:593–603. 26227898 10.1111/jeb.12331

[ece31886-bib-0033] Ronquist, F. , M. Teslenko , P. van der Mark , D. L. Ayres , A. Darling , S. Hohna , et al. 2012 MrBayes 3.2: efficient bayesian phylogenetic inference and model choice across a large model space. Syst. Biol. 61:539–542. 22357727 10.1093/sysbio/sys029 PMC3329765

[ece31886-bib-0034] Schoen, D. J. , and A. H. Brown . 1991 Intraspecific variation in population gene diversity and effective population size correlates with the mating system in plants. Proc. Natl Acad. Sci. USA 88:4494–4497. 11607182 10.1073/pnas.88.10.4494 PMC51687

[ece31886-bib-0035] Settepani, V. , J. Bechsgaard , and T. Bilde . 2014 Low genetic diversity and strong but shallow population differentiation suggests genetic homogenization by metapopulation dynamics in a social spider. J. Evol. Biol. 27:2850–2855. 25348843 10.1111/jeb.12520

[ece31886-bib-0036] Slotte, T. , J. P. Foxe , K. M. Hazzouri , and S. I. Wright . 2010 Genome‐wide evidence for efficient positive and purifying selection in *Capsella grandiflora*, a plant species with a large effective population size. Mol. Biol. Evol. 27:1813–1821. 20194429 10.1093/molbev/msq062

[ece31886-bib-0037] Stebbins, G. L. 1957 Self fertilization and population variability in the higher plants. Am. Nat. 91:337–354.

[ece31886-bib-0038] Supek, F. , and K. Vlahovicek . 2004 INCA: synonymous codon usage analysis and clustering by means of self‐organizing map. Bioinformatics 20:2329–2330. 15059815 10.1093/bioinformatics/bth238

[ece31886-bib-0039] Takebayashi, N. , and P. L. Morrell . 2001 Is self‐fertilization an evolutionary dead end? Revisiting an old hypothesis with genetic theories and a macroevolutionary approach. Am. J. Bot. 88:1143–1150. 11454614

[ece31886-bib-0040] Tonini, J. , A. Moore , D. Stern , M. Shcheglovitova , and G. Ortí . 2015 Concatenation and Species Tree Methods Exhibit Statistically Indistinguishable Accuracy under a Range of Simulated Conditions. *PLOS Currents Tree of Life*, Mar 9. Edition 1. 10.1371/currents.tol.34260cc27551a527b124ec5f6334b6be PMC4391732 25901289

[ece31886-bib-0041] Wright, S. 1931 Evolution in mendelian populations. Genetics 16:0097–0159. 10.1093/genetics/16.2.97 PMC1201091 17246615

[ece31886-bib-0042] Wright, S. I. , B. Lauga , and D. Charlesworth . 2002 Rates and patterns of molecular evolution in inbred and outbred Arabidopsis. Mol. Biol. Evol. 19:1407–1420. 12200469 10.1093/oxfordjournals.molbev.a004204

[ece31886-bib-0043] Yang, Z. H. 2007 PAML 4: phylogenetic analysis by maximum likelihood. Mol. Biol. Evol. 24:1586–1591. 17483113 10.1093/molbev/msm088

