# Supplementary material for: Phylogenetic analysis suggests that sociality is associated with reduced effectiveness of selection
Source: Ecol Evol. 2016 Jan 8;6(2):469–77. doi: 10.1002/ece3.1886 (PMC4729245; doi:10.1002/ece3.1886)
Supplement: Supplementary file 1 — Table S1. Primers designed for the phylogeny construction. Table S2. Substitution models estimated in Partition Finder (Lanfear et al. 2012) for each locus. Table S3. Inferred number of synonymous and non‐synonymous substitutions across each external branch. Figure S1. Base composition estimates in social and subsocial species of the genus Stegodyphus. Figure S2. Phylogeny including undescribed species Stegodyphus sp. [file ECE3-6-469-s001.docx]

**Supporting information**

**Table 1.** Primers designed for the phylogeny construction.

| **Primer** | **Forward primer’s sequence 5' - 3'** | **Reverse primer’s**  **Sequence 5' - 3'** | **Exon** | **Intron** | **Product length (bp)** | **Missing data** |
| --- | --- | --- | --- | --- | --- | --- |
| 5F-R | GTTCAGCAAGCAGATAAAGTSG | AGACAGTATTGWGAGGTTGC | 1 | 0 | 147 | *Eresus sandaliatus* |
| 9F-R | CAGTWGAGTTAGAACATCCTCG | RTCGTGAAACWGTRAGGC | 1 | 0 | 306 |  |
| 17F-R | GTCTGAGCAAYAACCGYGGC | GTTCCTCTATCAATGCCAGRG | 1 | 0 | 483 |  |
| 23F-R | GYTTCATACCTCCTGATGGTG | CATACAAKCCACTTCTTCC | 1 | 0 | 435 |  |
| 28F-R | GAAGGCAATGGAAGGTGGACG | CCAAAATACAATAACTGCTCTG | 1 | 0 | 420 |  |
| 40F-R | ACTGCTGCTCTTGGTCTCC | GACTGAGCAATCRTRTCCAGAG | 2 | 1 | 393 |  |
| 54F-R | CTGAAGAGCATAGGAACGGC | CCGTCWTCTGTCTGTSTAGTTGG | 2 | 1 | 461 |  |
| 58F-R | GTCCAAGCHAAAGCACAGG | TAGCTGCATTCTGAACCTGAG | 1 | 0 | 489 | *Adonea. fimbriata* |
| 69F-R | TGGCCTTTCCAYCAGTC | GKACATTGATATTCACCTTCAG | 1 | 0 | 348 |  |
| 102F-R | GAGGCCGACATGATATGCG | GGGCCATTCATCTGCTGAG | 1 | 0 | 681 |  |
| 109F-R | CAGACTCATCTACATGTTGTC | TGTATATCATCCAACTTRCAAC | 1 | 0 | 642 |  |
| 208F-R | CTGGTTCACGGTATGAAAC | GTTCCAGGCTTCTGRTTA | 1 | 1 | 153 | Intron: *Stegodyphus sp.* |
| 212F-R | GATTTCCTGGATATGATGCTG | TGATAGCAGCATGAGCTTTC | 2 | 1 | 380 |  |

**Table 2.** Substitution models estimated in Partition Finder (Lanfear et al 2012) for each locus.

| **Best model** | **Loci** |
| --- | --- |
| K80+G | 17F, 40F_Exons, 5F |
| HKY | 208F_Intron, 40F_Intron |
| HKY+G | 109F, 208F_Exon, 54F_Exons, 102F, 58F |
| HKY+I+G | 23F, 28F |
| GTR+G | 212F_Exon2, 212F_Intron, 54F_Intron |
| GTR+I | 212F_Exon1, 69F, 9F |

**Table 3.** Inferred number of synonymous and non-synonymous substitutions across each external branch. S = Number of synonymous positions, N = number of non-synonymous positions, dN/dS, dS = rate of synonymous positions, dN = rate of non-synonymous positions, S*dS = actual number of synonymous substitutions, N*dN = actual number of non-synonymous substitutions.

| External branch | S | N | dN/dS | dS | dN | S*dS | N*dN |
| --- | --- | --- | --- | --- | --- | --- | --- |
| *S. sarasinorum* | 880.4 | 2851.6 | 0. 0949 | 0.0111 | 0.0011 | 9.7 | 3.0 |
| *S. dumicola* | 880.4 | 2851.6 | 1.2489 | 0.0028 | 0.0035 | 2.5 | 10.1 |
| *S. mimosarum* | 880.4 | 2851.6 | 0.1986 | 0.0230 | 0.0047 | 20.3 | 13.3 |
| *S. bicolor* | 880.4 | 2851.6 | 0.0359 | 0.0098 | 0.0004 | 8.6 | 1.0 |
| *S. tibialis* | 880.4 | 2851.6 | 0.0709 | 0.0628 | 0.0046 | 55.3 | 13.2 |
| *S. pacificus* | 880.4 | 2851.6 | 0.0958 | 0.0073 | 0.0007 | 6.4 | 2.0 |
| *S. dufuori* | 880.4 | 2851.6 | 0.1445 | 0.0073 | 0.0011 | 6.4 | 3.0 |
| *S. africanus* | 880.4 | 2851.6 | 0.1677 | 0.0331 | 0.0055 | 29.2 | 15.7 |
| *S. tentoriicola* | 880.4 | 2851.6 | 0.2046 | 0.0112 | 0.0023 | 9.9 | 6.4 |
| *S. lineatus* | 880.4 | 2851.6 | 0.0930 | 0.1257 | 0.0119 | 110.7 | 33.8 |

**Figure 1.** Base composition estimates in social and subsocial species of the genus *Stegodyphus*.


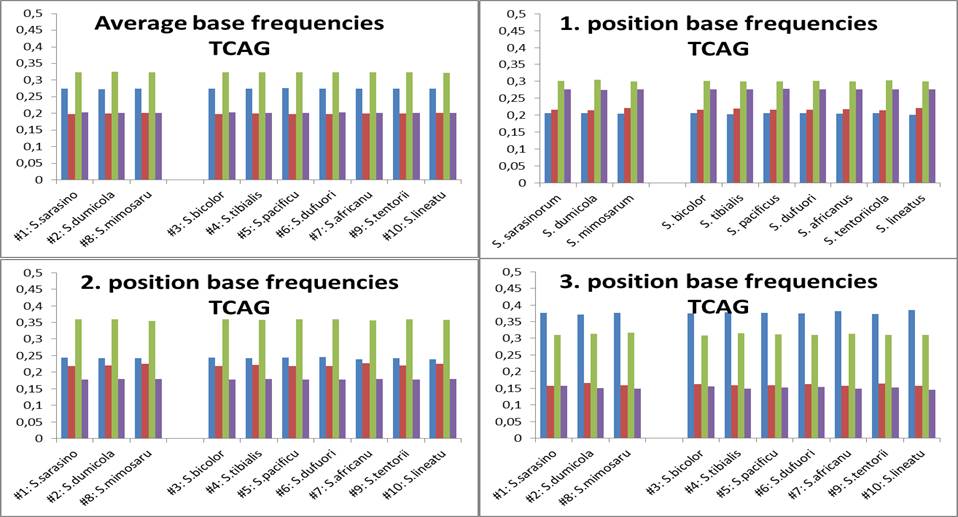


**Figure 2.** Phylogeny including undescribed species *Stegodyphus* sp. Bayesian phylogeny of the *Stegodyphus* genus including an undescribed species (*Stegodyphus* sp.). Posterior probabilities are shown for each node. Red branches lead to social species which are underlined.


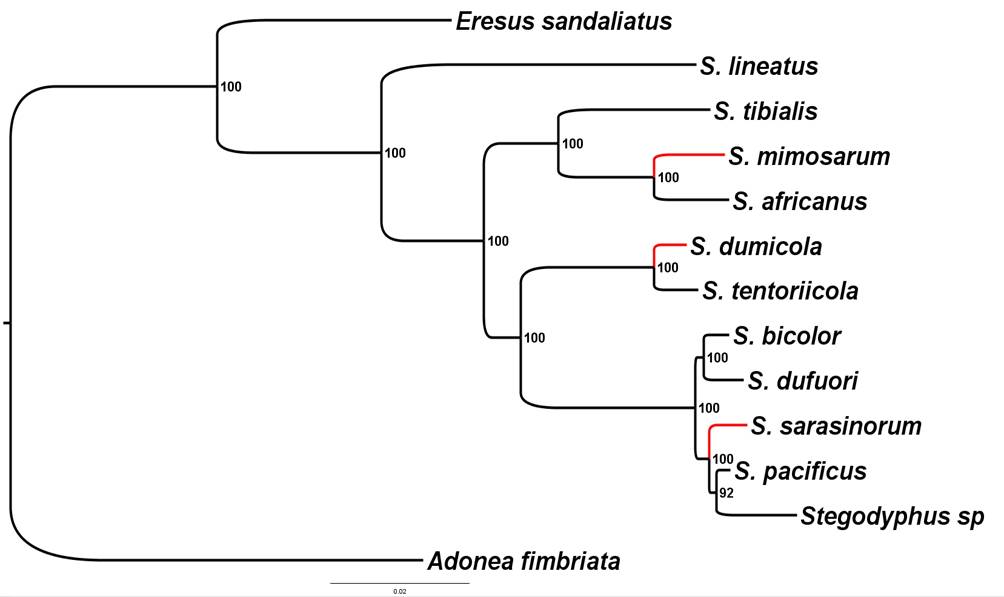


**References**

Lanfear R, Calcott B, Ho SYW, & Guindon S (2012) PartitionFinder: combined selection of partitioning schemes and substitution models for phylogenetic analyses. *Mol Biol Evol* 29(6):1695-1701.
